# Predictors of quality of life among patients on dialysis in southern Brazil

**DOI:** 10.1590/S1516-31802008000500002

**Published:** 2008-09-04

**Authors:** Maristela Bohlke, Diego Leite Nunes, Stela Scaglioni Marini, Cleison Kitamura, Marcia Andrade, Maria Paula Ost Von-Gysel

**Keywords:** Quality of life, Epidemiologic factors, End-stage renal disease, Dialysis, Risk, Qualidade de vida, Fatores epidemiológicos, Falência renal crônica, Diálise, Risco

## Abstract

**CONTEXT AND OBJECTIVE:**

Quality of life (QoL) is considered important as an outcome measurement, especially for long-term diseases such as chronic renal failure. The present study searched for predictors of QoL in a sample of patients undergoing dialysis in southern Brazil.

**DESIGN AND SETTING:**

This was a cross-sectional study developed in three southern Brazilian dialysis facilities.

**METHODS:**

Health-related QoL of patients on hemodialysis or peritoneal dialysis was measured using the generic Short Form-36 (SF-36) health survey questionnaire. The results were correlated with sociodemographic, clinical and laboratory variables. The analysis was adjusted through multiple linear regression.

**RESULTS:**

A total of 140 patients were assessed: 94 on hemodialysis and 46 on peritoneal dialysis. The mean age was 54.2 ± 15.4 years, 48% were men and 76% were white. The predictors of higher (better) physical component summary in SF-36 were: younger age (β -0.16; 95% confidence interval, CI: -0.27 to -0.05), shorter time on dialysis (β -0.06; 95% CI: -0.09 to -0.02) and lower Khan comorbidity-age index (β 5.16; 95% CI: 1.7-8.6). The predictors of higher mental component summary were: being employed (β 8.4; 95% CI: 1.7-15.1), being married or having a marriage-like relationship (β 4.56; 95% CI: 0.9-8.2), being on peritoneal dialysis (β 4.9; 95% CI: 0.9-8.8) and not having high blood pressure (β 3.9; 95% CI: 0.3-7.6).

**CONCLUSIONS:**

Age, comorbidity and length of time on dialysis were the main predictors of physical QoL, whereas socioeconomic issues especially determined mental QoL.

## RESUMO

**CONTEXTO E OBJETIVO:**

A qualidade de vida (QoL) é considerada uma importante medida de desfecho, especialmente no manejo de doenças de longa evolução, como a doença renal crônica. O presente estudo avaliou os preditores de QoL em uma amostra de pacientes tratados por diálise.

**TIPO DE ESTUDO E LOCAL:**

Estudo transversal com coleta de dados em três centros de diálise do sul do Brasil.

**MÉTODOS:**

Avaliação de pacientes tratados por diálise, com aplicação do questionário genérico SF-36 (Short Form-36) e correlação com variáveis demográficas, socioeconômicas, clínicas e laboratoriais. A análise foi ajustada por regressão linear múltipla.

**Resultados:**

Foram avaliados 140 pacientes, 94 tratados por hemodiálise e 46 por diálise peritoneal, com idade 54.2 ± 15,4 anos, 48% homens e 76% brancos. A dimensão física do SF-36 esteve associada à idade (β -0,17 intervalo de confiança, IC 95% -0,27-0,58), ao tempo em diálise (β -0,05 IC 95% -0,08-0,01) e ao índice de idade-comorbidade de Khan mais elevado (β 5,52 IC 95% 2,1-8,9). A dimensão mental esteve associada a ocupação remunerada (β 8,4 IC 95% 1,7-15,1), união estável (β 4,56 IC 95% 0,9-8,2), tratamento por diálise peritoneal (β 4,9 IC 95% 0,9-8,8) e ao fato de não ser hipertenso (β 3,9 IC 95% 0,3-7,6).

**CONCLUSÕES:**

Os pacientes mais jovens, tratados por diálise há menos tempo e com um menor número de comorbidades apresentaram melhor QoL do ponto de vista físico. A QoL mental foi melhor em pacientes que desempenham atividade remunerada, são casados, tratados por diálise peritoneal e não-hipertensos.

## INTRODUCTION

The incidence of end-stage renal disease (ESRD) has been increasing progressively all over the world, both in developed and in developing countries. In Brazil, a census promoted by the Brazilian Society of Nephrology (*Sociedade Brasileira de Nefrologia*, SBN) in January 2005 gathered data from 83% of the national dialysis facilities, showing a total of 54,311 patients undergoing dialysis: 48,362 of them on hemodialysis and the others on peritoneal dialysis.^1^ The costs involved in treating ESRD are very high, imposing great difficulties on public health systems, particularly in countries with limited resources, like Brazil. Despite the difficulties, all the renal replacement methods may be funded by the Brazilian Health Ministry and offered to every patient diagnosed with ESRD, without age or primary renal disease discrimination.

Despite the economic burden, the availability of renal replacement therapy maintains the lives of patients who otherwise would have succumbed to uremia. The length of survival obtained with hemodialysis or peritoneal dialysis is similar.^2^ However, survival is not the only expected result. According to Socrates, "*We should place the highest value not on living, but on living well."*

Quality of life (QoL) is increasingly being considered important as an outcome measurement. Therefore, the effect of different therapeutic options on QoL should necessarily be considered in healthcare planning. Among the treatment options for ESRD, kidney transplantation clearly produces the best results, both in terms of survival and in terms of QoL.^3,4^ However, kidney transplantation is not a realistic possibility for most ESRD patients, either because of the patient's own clinical conditions or because of insufficient availability of organs. Faced with this reality, a great number of patients end up undergoing dialysis for long periods. In order to offer the best QoL possible to this population, it is essential to determine the predictors of QoL for patients on dialysis. The relationship between patients' characteristics and QoL may be subject to a series of influences, including cultural ones.

## OBJECTIVE

The present study was designed to search for predictors of QoL among sociodemographic and clinical factors, in a sample of patients on dialysis in southern Brazil.

## METHODS

### Sample

The sample size was calculated with the aim of ensuring a statistical power of 80% and a significance level of 5%, such that a 15% difference in Short Form-36 (SF-36) mental or physical component summaries between hemodialysis and peritoneal dialysis patients was taken to be clinically significant. This calculation resulted in a minimum sample of 20 patients on peritoneal dialysis and 40 patients on hemodialysis.

The sample was selected from three dialysis facilities in the southern Brazilian city of Pelotas. Patients under 18 years of age or using a given dialysis method for less than six months were excluded.

### Methodology

This was a cross-sectional study, with measurement of QoL using a generic questionnaire, the SF-36 health survey. The sociodemographic and clinical data were obtained directly from patients or from their medical records. The laboratory tests represented the average of the results obtained over the preceding three months. Previously trained medical students collected the data and applied the questionnaires. The interviews were held at a time when the patients were not hospitalized or undergoing dialysis, which could have produced some discomfort, thus influencing the patients' perception of their QoL.

The variables assessed were: age, gender, skin color, marital status, income, education level, employment status, method of dialysis, length of time on dialysis, hematocrit, albumin, potassium, phosphorus, calcium, Khan comorbidity-age index, sexual function, diabetes and high blood pressure.

The Khan comorbidity-age index determines the risk relating to a patient's disease, based on comorbidity (coexisting pathological conditions that are not directly related to the uremic state) and age, as described by Khan et al.^5^ The low-risk group consists of patients less than 70 years old without a comorbidity. The medium-risk group consists of patients between 70 and 80 years old; patients less than 80 years old with one or more of the following diseases: angina, myocardial infarction, cardiac failure, chronic obstructive airway disease, pulmonary fibrosis, liver disease, peripheral vascular or cerebrovascular disease; and patients less than 70 years old with diabetes mellitus. The high-risk group consists of patients over 80 years of age; patients of any age with two or more organ dysfunctions in addition to ESRD; and patients of any age with visceral malignancy.

The instrument for measuring QoL was the SF-36. This is a generic questionnaire for measuring QoL that has been validated for the Portuguese language^6^ and has been used to assess a series of chronic diseases, including ESRD.^7,8^ This instrument evaluates patients' perceptions of their health-related QoL on a scale that goes from zero (complete dissatisfaction) to 100 (full satisfaction), involving eight domains: physical functioning (assessing the patient's capacity to respond to his/her physical needs, such as walking, running and going upstairs); physical role (evaluating how much the physical capacity limits the patient's activities); bodily pain (measuring the pain perception over the last four weeks and how much this pain has interfered with the physical activities); general health (measuring the patient's perception of his/her general health condition); vitality (evaluating the feelings of energy and fatigue); social functioning (evaluating how much the patient's physical health or emotional problems have interfered with social relationships, over the last four weeks); emotional role (measuring how much the patient's emotional factors have interfered in his/her job and in other activities); and mental health (evaluating the perception of anxiety and depression). These domains are divided in two component summaries: physical (physical functioning, physical role and bodily pain) and mental (social functioning, emotional role and mental health). The domains of general health and vitality are considered to belong to both component summaries.

### Statistical analysis

The raw and adjusted analyses of the SF-36 scores were performed using multiple linear regression. The SF-36 physical and mental component summaries were used as dependent variables and the sociodemographic and clinical variables were used as independent variables. In the event of finding statistically significant associations between the component summaries and any of the independent variables, the domains that constituted the component summaries were analyzed in a second stage, in order to identify which domains specifically contributed towards the final result. The adjustment model for linear regression was of backwards type, i.e. all the variables were included in the initial model and, through successive exclusions, only the variables that showed a value of p < 0.05 were kept in the model. The statistical software used was Stata 8.0 (Stata Corporation, College Station, Texas, United States).

### Ethical matters

The Ethics Committee of Universidade Católica de Pelotas approved the study and all the patients involved signed a written informed consent statement.

## RESULTS

A total of 140 patients were evaluated: 94 undergoing hemodialysis and 46 on peritoneal dialysis. Thus, the calculated minimum sample was exceeded. The characteristics of the sample are presented in Table 1 and the scores obtained in the SF-36 health survey are in Table 2.

**Table 1:** Patients' characteristics (n = 140)

Age (years) (mean ± SD)	54.2 ± 15.4
Male (n)	67 (48%)
White (n)	107 (76%)
Marital status (n)	
Single	32 (23%)
Married	72 (51%)
Widowed	23 (17%)
Separated	13 (9%)
Duration of schooling ≤ 8 years (n)	113 (81%)
In employment (n)	11 (8%)
Hemodialysis (n)	94 (67%)
Length of time on dialysis (months) (median/range)	46 (6-264)
Monthly household income (R$)* (median/range)	520 (0-5,200)
Hematocrit (%)	29.8 ± 5.5
Albumin	3.6 ± 0.5
Potassium	5.1 ± 1
Phosphorus	6.3 ± 2.1
Calcium	10.1 ± 1.4
Comorbidity-age index (n)	
Low	49 (35%)
Medium	78 (56%)
High	13 (9%)
Diabetes (n)	25 (18%)

SD = standard deviation; R$ = Brazilian Real.

* Value in United States dollars: US$ 289 as in May, 2007 (US$0,00 – US$2,889,00).

**Table 2:** Short Form-36 (SF-36) domain scores and summary measurements (mean ± standard deviation, SD) among 140 patients under dialysis

Physical component summary	44.5 ± 10.3
Mental component summary	51.2 ± 11.3
Physical functioning	65.4 ± 32.7
Physical role	68.7 ± 38.8
Bodily pain	74.8 ± 31.6
General health	60.5 ± 24.5
Vitality	69.3 ± 25.3
Social functioning	78.7 ± 30.2
Emotional role	74.7 ± 39.4
Mental health	72 ± 21.9

### Univariate analysis

In the univariate analysis, higher scores in the physical component summary were found among patients who were younger (p < 0.001), had been on dialysis for shorter lengths of time (p = 0.02), had a lower comorbidity-age index (p < 0.001), had lower education (p = 0.02), were in employment (p = 0.05) and had no sexual dysfunction (p = 0.009). In the mental component summary, higher scores were presented by patients who were on peritoneal dialysis (p = 0.05), were not hypertensive (p = 0.003), were married or in a marriage-like relationship (p = 0.03) and were in employment (p = 0.01).

Among the domains that constitute the SF-36 physical and mental component summaries, higher physical functioning scores were associated with younger age (p < 0.001), lower Khan index (p = 0.01), being single (p = 0.03), having lower education (p = 0.03), having a job (p = 0.01) and not having sexual dysfunction (p = 0.003). The best scores in the physical role domain were found among individuals who had been on dialysis for shorter times (p = 0.05), were younger (p = 0.006), had lower Khan index (p = 0.05), were in employment (p = 0.04), had good sexual function (p = 0.01) and had higher hematocrit (p = 0.04). The bodily pain scores were higher among patients who were on peritoneal dialysis (p = 0.003), had lower Khan index (p = 0.01) and had been on dialysis for shorter times (p = 0.007). The general health scores were better among individuals who had been on dialysis for shorter times (p = 0.008) and had lower Khan index (p = 0.01). The vitality scores were higher among individuals who were younger (p = 0.002), had lower Khan index (p = 0.001), were single (p = 0.04) and were in employment (p = 0.003). Higher social functioning scores were associated with individuals who were younger (p = 0.001), had lower Khan index (p = 0.004) and were in employment (p = 0.04). The emotional role scores were higher among patients who were on peritoneal dialysis (p = 0.02), were not hypertensive (p < 0.001), were not diabetic (p = 0.008), were married (p = 0.03) and had higher income (p = 0.04). Better mental health was detected among married individuals (p = 0.03), individuals who had higher income (p = 0.04) and those who had a job (p = 0.004).

### Multivariate analysis

Younger age, shorter time on dialysis and lower Khan comorbidity-age index were predictors of higher scores in the SF-36 physical component summary, even after adjustments through multivariate linear regression (Table 3). The independent predictors of higher mental component summary were having a job, being married or having a marriage-like relationship, not being hypertensive and being on peritoneal dialysis (Table 3).

**Table 3:** Adjusted analysis for predictors of physical and mental component summaries of the Short Form (SF-36) among 140 patients under dialysis

	Coefficients (95% CI)	p-value*
**Physical component summary**		
Age	-0.16 [-0.27-(-0.05)]	0.004
Comorbidity-age index	-5.16 [-8.61-(-1.71)]	0.004
Length of time on dialysis	-0.06 [-0.09-(-0.02)]	0.002
**Mental component summary**		
Hemodialysis versus peritoneal dialysis	-4.89 [-8.8-(-0.98)]	0.01
High blood pressure	-3.97 [-7.64-(-0.3)]	0.03
Married	4.56 (0.95-8.18)	0.01
In employment	8.4 (1.7-15.1)	0.01

* Wald test; CI = confidence interval.

Among the domains that constitute the SF-36 physical component summary, age played a significant influence on physical functioning, physical role and vitality scores. The length of time on dialysis was associated especially with the physical role, bodily pain and general health scores. The Khan comorbidity-age index was significantly associated with the physical functioning and general health scores (Table 4).

**Table 4:** Adjusted analysis for predictors of Short Form-36 (SF-36) domains among patients under dialysis

	Coefficients (95% CI)	p-value*
**Physical functioning**		
Age	-0.7 [-1.05-(-0.36)]	< 0.001
Comorbidity-age index	-12.5 [-23.4-(-1.6)]	0.02
**Physical role**		
Age	-0.48 [-0.91-(-0.05)]	0.03
Length of time on dialysis	-0.19 [-0.34-(-0.04)]	0.01
**Bodily pain**		
Length of time on dialysis	-0.18 [-0.3-(-0.05)]	0.006
**General health**		
Comorbidity-age index	-11.6 [-20.7-(-2.50]	0.01
Length of time on dialysis	-0.13 [-0.22-(-0.03)]	0.01
**Vitality**		
Age	-0.45 [-0.74-(-0.170]	0.002
In employment	23.27 (7.76-38.78)	0.004
**Emotional role**		
Hemodialysis	-21.4 [-34.7-(-8.04)]	0.002
High blood pressure	-21.6 [-34.2-(-9.1)]	0.001
Married	16.1 (3.76-28.42)	0.01
**Mental health**		
Married	8.46 (1.4-15.5)	0.02
In employment	18.58 (5.5-31.6)	0.006

* Wald test; CI = confidence interval.

Concerning the domains that constituted the SF-36 mental component summary, employment had significant associations with vitality and mental health. Marriage was associated with better mental health scores. Patients undergoing peritoneal dialysis presented higher scores in the domain of emotional role and a tendency towards higher mental health scores. Hypertensive patients presented lower emotional role scores (Table 4).

No associations were detected between any other sociodemographic or clinical variables and the scores of the SF-36 questionnaire.

## DISCUSSION

The domains that make up the physical QoL were more impaired than were the domains that constitute the mental QoL, which appeared to be closer to scores from general populations in several countries. This finding is in line with the results obtained from other studies, which demonstrated poorer physical QoL in relation to mental QoL, in the population on dialysis.^7,8^ This may reflect the ability of ESRD patients to adapt psychologically to their situation over time. A report on patients who had recently started dialysis described mental scores that were at the level of mild depression.^9^ Mittal et al. demonstrated that, after the first months of dialysis, there was an improvement in the mental QoL.^10^ Other studies have reported decreases in the physical aspects of QoL over time among patients on dialysis, but the effects of time in the mental QoL seemed not to be significant.^10,11^

In the present study, younger age, shorter time on renal replacement therapy and fewer comorbidities predicted better physical QoL. In other studies, comorbidities clearly influenced the physical QoL, both among patients on conservative treatment^12,13^ and among those on dialysis.^14^ The presence of diabetes was especially associated with worse QoL among ESRD patients,^11,14,15^ although this association was not detected in the sample of the present study.

The physical QoL in the general population starts to decline from the fifth decade of life onwards. Among older ESRD patients, the physical QoL is usually more impaired and the mental QoL is closer to that of the general population in the same age group.^16^ In the present sample, higher mental QoL scores were predicted by the characteristics of being employed, maintaining a stable relationship, being on peritoneal dialysis and not being hypertensive. Kusek et al. also detected that marital and employment status presented an association with the mental component summary of SF-36, in a study among ESRD patients of black race.^13^

The degree of support received within the family environment has been described as an important predictor of mental QoL among ESRD patients.^17^ However, the effects of the family's involvement are not always shown to be beneficial for the patient, and they may vary between the extremes of not giving any assistance and taking control over the patient's life.^10^

Previous studies have also demonstrated that the type of treatment has an influence on the QoL of ESRD patients. The patients undergoing kidney transplantation clearly experience the best results, both in physical and in mental QoL.^11^ Concerning the different types of dialysis, there are no well-established differences in QoL, although some studies describe advantages in peritoneal dialysis, especially regarding the mental quality of life.^7^ However, in none of these studies were the patients randomized with regard to different types of dialysis. Therefore, it is not possible to rule out the possibility of selection bias. In the present study, which was also non-randomized, treatment with peritoneal dialysis remained significantly associated with better mental QoL, even after adjusted analysis. It is possible, however, that variables that were not analyzed may have been influencing the differences found. Perhaps treatment selection rather than the treatments themselves influenced the mental process scale differences between the groups. Independent people, for example, with a positive and assertive outlook and a high internal locus of control might have chosen peritoneal dialysis because of the nature of that treatment.

Despite the small number of our patients who were in employment, having a job was an important predictor of better mental QoL. Holding down a job certainly has a positive influence on the perception that an individual has of his or her role in society and it contributes towards improved self-esteem, which is considered to be an important aspect of QoL. Becoming unemployed increases the burden attributed to renal disease, especially if the patient was the primary provider for the family. In the present study, having a job remained associated with mental QoL even after adjustment for income level. Therefore, the role of a job in relation to QoL seems to transcend financial matters. Among the sample studied, the patients without a job were not subdivided between those who had voluntarily withdrawn from the job market and those who had really become unemployed. However, it is known that the unemployment rate among dialysis populations is much higher than the rate among the general population.^18^

None of the laboratory variables analyzed was associated significantly with physical or mental QoL. Similar findings have been described by other authors.^17^ Older studies reported an association between higher hematocrit and QoL.^19^ These studies, however, were conducted at a time when the use of erythropoietin was restricted and dialysis patients presented lower hematocrit. Albumin is a known predictor of morbidity and mortality in dialysis populations, and many studies have also associated higher albumin levels with better QoL.^10,20^ This association was not detected in the present study, possibly because the albumin values in this sample were relatively homogeneous.

Because of the great number of statistical comparisons made, the possibility that some associations appeared by chance cannot be ruled out. However, most of the associations detected have also been described in previous studies involving similar populations.

## CONCLUSIONS

Considering the importance of QoL as a direct outcome measurement, in addition to its influence on other measurements like morbidity and mortality,^8^ identification of predictors of better QoL may make it possible to intervene in relation to modifiable factors, such as establishing policies that could increase the integration of ESRD patients in the job market. In addition to the QoL benefits for such patients, their involvement with productive activities would result in a gain for all of society, with a reduction in the burden of maintaining inactive individuals.
